# Incidence of Placebo Adverse Events in Randomized Clinical Trials of Targeted and Immunotherapy Cancer Drugs in the Adjuvant Setting: A Systematic Review and Meta-analysis

**DOI:** 10.1001/jamanetworkopen.2018.5617

**Published:** 2018-12-07

**Authors:** Matías Rodrigo Chacón, Diego Hernán Enrico, Jeannette Burton, Federico Daniel Waisberg, Viviana Marina Videla

**Affiliations:** 1 Research Department, Argentine Association of Clinical Oncology, Buenos Aires, Argentina

## Abstract

**Importance:**

Several reports have associated the placebo effect with objective response and improvement of a clinical condition in oncology, but only a few studies have analyzed the adverse events (AEs) in the placebo groups of the clinical trials.

**Objective:**

To determine the incidence of placebo AEs reported in randomized clinical trials of modern cancer drugs in the adjuvant setting.

**Data Sources:**

Based on the Preferred Reporting Items for Systematic Reviews and Meta-analyses (PRISMA) reporting guideline, a systematic literature search of English-language publications from January 1, 2000, through April 15, 2018, was performed using MEDLINE (PubMed). The following search terms were used to retrieve all trials from the PubMed library: *adjuvant*, *maintenance*, *consolidation*, and *placebo*, in addition to specific cancer type–related keywords.

**Study Selection:**

A double-blind, randomized, placebo-controlled, phase 3 design was mandatory for study inclusion. Only studies enrolling patients who had undergone macroscopically complete resections were included. No other anticancer treatments in addition to placebo were allowed in the control group. Only trials involving a targeted therapy (tyrosine kinase, BRAF, or MEK inhibitors) or immunotherapy-related drugs were included. Trials using chemotherapy, interferon, and endocrine therapy were excluded. Two authors (D.H.E. and F.D.W.) independently reviewed the studies for inclusion.

**Data Extraction and Synthesis:**

Data were extracted by investigators, and random-effects meta-analysis was performed to estimate the proportion of grade 3 to 4 placebo AEs in the included studies.

**Main Outcomes and Measures:**

Incidence of grade 3 to 4 placebo AEs in the placebo groups.

**Results:**

Of 731 studies screened, 10 eligible trials were found including 4 tumor types (melanoma, non–small cell lung cancer, gastrointestinal stromal tumor, and renal cell carcinoma). Overall, 11 143 patients (6270 [56.3%] in the treatment group with mean [SD] age of 55.6 [4.2] years and 4873 patients [43.7%] in the placebo group with mean [SD] age of 55.9 [4.3] years) were included. The mean incidence of any-grade placebo AEs was 85.1% (95% CI, 79.2%-91.0%). The most frequent (mean [SD]) grade 3 to 4 placebo AEs in patients were hypertension (2.8% [2.2%]), fatigue (1.0% [0.9%]), and diarrhea (0.8% [0.6%]). The overall, random-effects pooled incidence of grade 3 to 4 placebo AEs was 18% (95% CI, 15%-21%), with a high level of heterogeneity (*I*^2^ = 86%). Frequency of grade 3 to 4 placebo AEs was found to be correlated in the treatment and placebo groups (ρ = 0.7; *P* = .03). Mean study drug discontinuation owing to placebo AEs was 3.9% (95% CI, 2.7%-5.2%).

**Conclusions and Relevance:**

Placebo administration was associated with a substantial incidence of grade 3 to 4 placebo AEs in modern cancer adjuvant trials. This finding should be considered by investigators, sponsors, regulatory authorities, and patient support groups.

## Key Points

**Question:**

What is the incidence of grade 3 to 4 adverse events in the placebo groups of modern cancer drug trials in the adjuvant setting?

**Findings:**

In this systematic review and meta-analysis including 11 143 patients and 10 adjuvant, double-blind, randomized, placebo-controlled, phase 3 trials, the incidence of grade 3 to 4 adverse events in the placebo groups was 18%.

**Meaning:**

This finding suggests that placebo administration may be associated with a substantial incidence of grade 3 to 4 adverse events in modern cancer adjuvant trials and should be considered by investigators and patients.

## Teaser

This systematic review and meta-analysis examines randomized clinical trials (RCTs) of modern cancer drugs in the adjuvant setting to determine the incidence of severe adverse events reported in placebo groups.

## Introduction

The placebo phenomenon refers to a patient’s symptom improvement when receiving an inert agent or procedure. Placebos have been associated not only with patient comfort but also with beneficial responses (placebo effect) at least since the 18th century.^1,2^ There are different explanations about this phenomenon, including neurobiological pathways, the physician-patient relationship, and the patient’s psychosocial context.^3^

Adverse events (AEs) resulting from placebo administration are called *nocebo effects*.^4,5,6^ These AEs have been studied mainly in analgesia, dermatology, psychiatry (depression), and neurology.^7,8,9,10^ In oncology, there are several studies about the placebo effect associated with objective response and improvement of clinical conditions, but there are only a few about placebo AEs.^11,12,13^

Even though studies found that approximately 25% of randomized patients reported placebo AEs that could be severe enough to lead to trial discontinuation, it was acknowledged that three-quarters of health care professionals and patients were unaware of the existence of placebo AEs.^6,14,15,16^ The type and incidence of AEs seem to be similar in both groups (treatment and placebo) among different randomized clinical trials (RCTs). These similarities may be attributable to the communication of potential AEs in the informed consent.^6,17,18^

Although it is expected that patients report mostly low-grade AEs after surgical treatments or radiation therapy, impairing symptoms such as moderate to severe fatigue are described in the literature.^19,20^ These factors, as well as recurrence-related symptoms, can also contribute to the toxic effects analyzed during trials in the adjuvant setting.

The aim of this study was to determine the incidence of placebo AEs reported in RCTs of modern cancer drugs. Only trials that included patients in the adjuvant setting were considered to exclude AEs caused by an untreated or persistent disease.

## Methods

### Literature Search Strategy

This meta-analysis search followed the Preferred Reporting Items for Systematic Reviews and Meta-analyses (PRISMA) reporting guideline.^21^ The systematic literature search was performed on April 15, 2018, using publications available in MEDLINE (PubMed). The final search was restricted to clinical trials and English-language publications from January 1, 2000, to the date of the search. The following search terms were used to retrieve all trials from the PubMed library: *adjuvant*, *maintenance*, *consolidation*, and *placebo*, in addition to specific cancer type–related keywords. The search strategy is detailed in the eMethods in the Supplement.

A double-blind, randomized, placebo-controlled, phase 3 design was mandatory for inclusion. No other anticancer treatments in addition to placebo were allowed in the control group. Only trials involving a targeted therapy (tyrosine kinase, BRAF, or MEK inhibitors) or immunotherapy-related drugs (agents that directly enhance immune antitumor activity) in the adjuvant setting were included. Trials using chemotherapy, endocrine therapy, or interferon were excluded because these drugs are associated with considerably different incidences and types of AEs, which might alter the interpretation of the results. Furthermore, trials that randomized patients to chemotherapy or hormonal therapy vs placebo in the adjuvant setting have been rare. Studies that included patients with any evidence of disease or enrolled patients before 2000, as well as studies without complete recruitment or with primary end points not associated with cancer treatment efficacy (eg, pharmacokinetics, biomarkers, or predictive factors research), were also excluded. Considering the heterogeneous criteria to determine this last statement among trials in an adjuvant setting, only studies including patients who had undergone macroscopically complete resections were included. Finally, trials with unavailable essential information, such as grade 3 to 4 AE rate, were excluded.

### Study Selection and Data Extraction

The trials were initially selected considering their titles and abstracts. Two investigators reviewed the abstracts for inclusion (D.H.E. and F.D.W.). Eligibility criteria were applied to the full-text articles during the final selection. The resulting list of included articles was discussed by all the investigators to ensure the accuracy of the final decision. Redundancy due to data reported on identical patient groups in different publications was analyzed, and duplications were removed. Data from the full text and the appendix were extracted, and critical information from each article was recorded onto predefined forms. The total number of patients in the safety analysis was collected from the included articles. The trial design, cancer types and stages, Common Terminology Criteria for Adverse Events (CTCAE) version, planned treatment duration, route of administration, and time from surgical resection to randomization were recorded. Comparisons were performed between treatment group (active therapy) and placebo group for grade 1 to 5 AEs, study drug discontinuation, dose reduction, study drug interruption, and duration of study drug exposure.

### Statistical Analysis

Frequencies and descriptive analyses were performed for each variable using SPSS (IBM), version 23.0. Meta-analysis was undertaken with a random-effects model conducted in the Meta package of R Studio Software, version 1.1.456 (RStudio). Heterogeneity among studies was assessed by *I*^2^ statistics. Results were reported as forest plots showing the proportion of grade 3 to 4 placebo AEs in the included studies with 95% CIs. Frequency of grade 3 to 4 AEs when comparing the treatment group with placebo group was analyzed by the Spearman rank correlation coefficient. The Cochrane Collaboration risk of bias tool was used independently by 2 investigators (M.R.C. and J.B.).^22^ Disagreements were solved by consensus. The risk of bias summary was performed using RevMan (Cochrane Collaboration), version 5.1. Publication bias was assessed using Egger tests with a funnel plot.^23^

## Results

### Search Results

**Figure 1.**
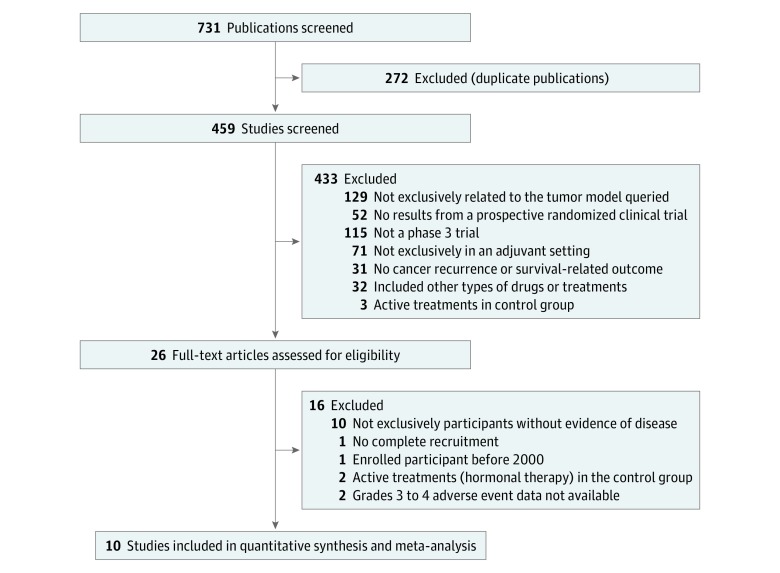
Study Selection Process of Randomized Clinical Trials of Modern Cancer Drugs

Using the defined search strategy, a total of 731 publications were obtained. The search flow is detailed in Figure 1. After duplication removal, all titles and abstracts of the resulting studies were screened. A full-text analysis was conducted for the resulting 26 articles, leading to 10 trials included for meta-analysis. Two trials were excluded from the study because information regarding grade 3 to 4 AEs was unavailable.^24,25^

### Included Studies

A total of 10 RCTs were considered for this review that included 4 tumor types: melanoma, 4 RCTs; non–small cell lung cancer, 1 RCT; gastrointestinal stromal tumor, 1 RCT; and renal cell carcinoma, 4 RCTs. Selected trials involved a total of 11 143 patients, including 6270 (56.3%) in the treatment groups (mean [SD] age, 55.6 [4.2] years) and 4873 (43.7%) in the placebo groups (mean [SD] age, 55.9 [4.3] years) (eTable 1 in the Supplement).

In the placebo groups, 64% of the participants included were men and 36% were women, whereas most patients included were white (89.8%). The majority of patients in the trials had an Eastern Cooperative Oncology Group performance status of 0 (76.9%), with a distribution of 22.3% with a performance status of 1 and 1.4% with a performance status of 2. A summary of data extracted from the selected studies,^26-35^ including the route of administration of the placebos, is given in Table 1. Additional information is available in eTable 2 in the Supplement.

**Table 1.** Characteristics of the 10 Selected Randomized Clinical Trials of Modern Cancer Drugs in the Adjuvant Setting

Study	Cancer Type	Treatment Group Drug	Route of Administration	Cancer Stage	CTCAE Version
Eggermont et al,^26^ 2015	Melanoma	Ipilimumab	Parenteral	III	3.0
Long et al,^27^ 2017	Melanoma	Dabrafenib and trametinib	Oral	III	4.0
Maio et al,^28^ 2018	Melanoma	Vemurafenib	Oral	IIC-IIIC	4.0
Eggermont et al,^29^ 2018	Melanoma	Pembrolizumab	Parenteral	III	4.0
Vansteenkiste et al,^30^ 2016	Non–small cell lung cancer	MAGE-A3	Parenteral	IB, II, IIIA	3.0
DeMatteo et al,^31^ 2009	Gastrointestinal stromal tumor	Imatinib	Oral	T≥3 cm	3.0
Haas et al,^32^ 2016	Renal cell carcinoma	Treatment group 1: sunitinib; treatment group 2: sorafenib	Oral	I-III	3.0
Motzer et al,^33^ 2017	Renal cell carcinoma	Pazopanib	Oral	II (high grade)-IV (M0)	4.0
Chamie et al,^34^ 2017	Renal cell carcinoma	Girentuximab	Parenteral	I-II (high grade), III-IV (M0)	NR
Ravaud et al,^35^ 2016	Renal cell carcinoma	Sunitinib	Oral	III-IV (M0)	3.0

Abbreviations: CTCAE, Common Terminology Criteria for Adverse Events; MAGE-A3, melanoma-associated antigen 3; NR, not reported.

### Study Outcomes

The characteristics of the selected studies comparing the treatment groups with placebo groups are detailed in Table 2. The mean incidence of any-grade placebo AEs was 85.1% (95% CI, 79.2%-91.0%). The frequency of grade 3 to 4 AEs in the treatment vs placebo groups among all trials is shown in eFigure 1 in the Supplement. The most frequent (mean [SD]) grade 3 to 4 placebo AEs were hypertension (2.8% [2.2%]), fatigue 1.0% [0.9%], and diarrhea (0.8% [0.6%]) (Table 3).

**Table 2.** Characteristics of the 10 Selected Randomized Clinical Trials of Modern Cancer Drugs Analyzing the Treatment Group vs the Placebo Group

Study	No. of Patients^a^	Median Study Drug Duration, mo	Patients Who Discontinued Study Drug, No. (%)		Patients With Adverse Events, No. (%)		Grade 5 Adverse Events, No.^b^
			Adverse Events	Disease Recurrence	Any Grade	Grade 3-4	
Eggermont et al,^26^ 2015							
Treatment group	471	3	245 (52)	132 (28)	465 (99)	254 (54)	5
Placebo group	474	15	20 (4)	273 (58)	431 (91)	118 (25)	0
Long et al,^27^ 2017							
Treatment group	435	11	114 (26)	23 (5)	422 (97)	180 (41)	1^c^
Placebo group	432	10	12 (3)	175 (41)	380 (88)	61 (14)	0
Maio et al,^28^ 2018							
Treatment group	247	12	49 (20)	26 (10)	245 (99)	141 (57)	0
Placebo group	247	12	5 (2)	87 (35)	219 (89)	37 (15)	0
Eggermont et al,^29^ 2018							
Treatment group	509	12	70 (14)	109 (21)	475 (93)	161 (32)	1
Placebo group	502	12	11 (2)	179 (36)	453 (90)	93 (19)	0
Vansteenkiste et al,^30^ 2016^d^							
Treatment group	1515	NR	120 (8)	555 (37)	1369 (90)	233 (15)	0
Placebo group	757	NR	54 (7)	271 (36)	556 (73)	114 (15)	0
DeMatteo et al,^31^ 2009							
Treatment group	337	NR	57 (17)	1 (0.3)	333 (99)	104 (31)	0
Placebo group	345	NR	11 (3)	41 (12)	314 (91)	63 (18)	0
Haas et al,^32^ 2016							
Treatment group 1^e^	625	11	124 (20)	52 (8)	NR	394 (63)	3
Treatment group 2^f^	628	11	128 (20)	54 (8)	NR	450 (72)	1
Placebo group	626	12	33 (5)	102 (16)	NR	159 (25)	0
Motzer et al,^33^ 2017							
Treatment group	766	10.5	278 (36)	47 (6)	755 (99)	469 (61)	1
Placebo group	762	NR	40 (5)	148 (19)	675 (89)	161 (21)	0
Chamie et al,^34^ 2017							
Treatment group	431	5.2	7 (2)	29 (7)	288 (67)	51 (12)	0
Placebo group	424	5.1	4 (1)	30 (7)	281 (66)	45 (11)	0
Ravaud et al,^35^ 2016							
Treatment group	306	12.4	86 (28)	22 (7)	305 (100)	185 (61)	0
Placebo group	304	12.4	17 (6)	59 (19)	269 (89)	59 (19)	0

Abbreviation: NR, not reported.

^a^ Patients included in safety analysis.

^b^ Drug related.

^c^ Not specified as drug related.

^d^ In both treatment and placebo groups, 52% of patients received adjuvant chemotherapy.

^e^ Sunitinib.

^f^ Sorafenib.

**Table 3.** Characteristics of Grade 3 to 4 Adverse Events in the Selected Randomized Clinical Trials of Modern Cancer Drugs^a^

Study	Total Patients, No.	Symptom-Driven Adverse Events, No. of Patients (%)					Nonsymptom-Driven Adverse Events, No. of Patients (%)						
		Fatigue	Nausea	Diarrhea	Vomiting	Abdominal Pain	Pyrexia	Rash	Hand-Foot Syndrome	ALT Increased	AST Increased	Hypertension
**Melanoma Studies**												
Eggermont et al,^26^ 2015												
Treatment group	471	10 (2.1)	1 (0.2)	46 (10)	2 (0.4)	2 (0.4)	5 (1)	6 (1)	NR	25 (5)	20 (4.2)	NR
Placebo group	474	7 (1.4)	0	9 (2)	1 (0.2)	1 (0.2)	1 (0.2)	0	NR	0	0	NR
Long et al,^27^ 2017												
Treatment group	435	19 (4)	4 (1)	4 (1)	4 (1)	NR	23 (5)	0	NR	16 (4)	16 (4)	25 (6)
Placebo group	432	1 (0.2)	0	1 (0.2)	0	NR	2 (0.5)	1 (0.2)	NR	1 (0.2)	1 (0.2)	8 (2)
Maio et al,^28^ 2018												
Treatment group	247	7 (3)	1 (0.4)	5 (2)	0	1 (0.4)	0	14 (5.4)	NR	14 (5.4)	8 (3)	6 (2)
Placebo group	247	1 (0.4)	0	2 (1)	0	0	0	3 (1)	NR	1 (0.4)	1 (0.4)	2 (1)
Eggermont et al,^29^ 2018												
Treatment group	509	4 (0.8)	0	4 (0.8)	NR	NR	NR	1 (0.2)	NR	NR	NR	NR
Placebo group	502	2 (0.4)	0	3 (0.6)	NR	NR	NR	0	NR	NR	NR	NR
**Non–Small Cell Lung Cancer Study**												
Vansteenkiste et al,^30^ 2016												
Treatment group	1515	7 (0.5)	NR	NR	NR	NR	3 (0.2)	NR	NR	NR	NR	NR
Placebo group	757	1 (0.1)	NR	NR	NR	NR	0	NR	NR	NR	NR	NR
**Gastrointestinal Stromal Tumor Study**												
DeMatteo et al,^31^ 2009												
Treatment group	337	7 (1.6)	8 (2)	10 (2)	8 (2)	12 (3)	NR	NR	NR	9 (2.6)	0	NR
Placebo group	345	4 (1)	4 (1)	5 (1)	2 (0.6)	6 (1)	NR	NR	NR	7 (1.9)	0	NR
**Renal Cell Carcinoma Studies**^b^												
Haas et al,^32^ 2016												
Treatment group 1^c^	625	110 (18)	23 (4)	62 (10)	14 (2)	NR	NR	15 (2)	94 (15)	NR	NR	105 (17.2)
Treatment group 2^d^	628	44 (7)	8 (1)	58 (9)	7 (1)	NR	NR	95 (15.3)	208 (33)	NR	NR	102 (16)
Placebo group	626	19 (3)	1 (0.2)	3 (0.5)	2 (0.3)	NR	NR	3 (0.5)	7 (1)	NR	NR	26 (4)
Motzer et al,^33^ 2017												
Treatment group	766	17 (2.2)	4 (0.5)	52 (6.8)	3 (0.4)	8 (1.1)	NR	1 (0.1)	19 (2.5)	120 (15.7)	46 (6)	197 (25.7)
Placebo group	762	5 (0.7)	0	7 (0.9)	3 (0.4)	1 (0.1)	NR	0	0	5 (0.6)	1 (0.1)	46 (6)
Ravaud et al,^35^ 2016												
Treatment group	306	15 (4.9)	6 (2)	12 (3.9)	7 (2.3)	5 (1.6)	1 (0.3)	2 (0.7)	49 (16)	NR	NR	24 (7.8)
Placebo group	304	4 (1.3)	0	1 (0.3)	0	1 (0.3)	0	0	1 (0.3)	NR	NR	4 (1.3)

Abbreviations: ALT, alanine aminotransferase; AST, aspartate aminotransferase; NR, not reported.

^a^ Patients included in the safety analysis.

^b^ No adverse events were reported for Chamie et al.^34^

^c^ Sunitinib.

^d^ Sorafenib.

**Figure 2.**
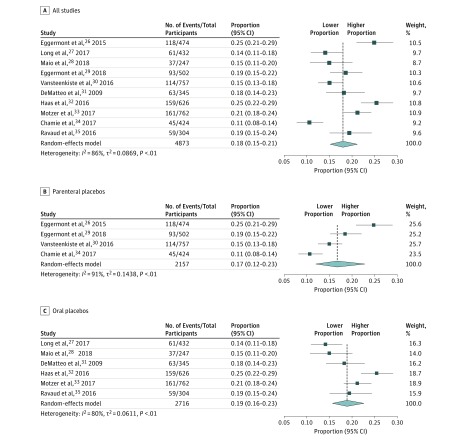
Proportion of Grade 3 to 4 Placebo Adverse Events in Randomized Clinical Trials of Modern Cancer Drugs The vertical line shows the overall effect. The data markers indicate the proportion of grade 3 to 4 adverse events for each study. The size of the data markers indicates the respective weight of the individual effects in the overall analysis. Error bars indicate 95% CIs.

The incidence of grade 3 to 4 toxic effects in the placebo groups of the selected trials was included in the meta-analysis.^26-35^ The overall, random-effects pooled incidence of grade 3 to 4 placebo AEs was 18% (95% CI, 15%-21%), with a high level of heterogeneity (*I*^2^ = 86%) (Figure 2). The incidence of grade 3 to 4 placebo AEs reached values higher than 20% in 3 trials. When the same analysis was performed analyzing the trials involving oral and parenteral placebos, the incidence of grade 3 to 4 placebo AEs was 19% (95% CI, 16%-23%) for oral placebos and 17% (95% CI, 12%-23%) for parenteral placebos. After subdividing by different tumor types, the incidence of grade 3 to 4 placebo AEs was 18% (95% CI, 14%-23%) in melanoma RCTs and 19% (95% CI, 14%-25%) in renal cell carcinoma RCTs, with high heterogeneity (*I*^2^ = 85% and 91% respectively) (eFigure 2 in the Supplement).

A positive correlation was observed in the frequency of grade 3 to 4 AEs between the treatment and placebo groups (ρ = 0.7; *P* = .03) (eFigure 3 in the Supplement). No deaths were reported to be associated with the placebo intervention. In the study by Vansteenkiste et al,^30^ 52% of patients received adjuvant chemotherapy before randomization in both treatment and placebo groups. Rates of grade 3 to 4 AEs in the 2 groups were equal.

Most trials reported a median placebo study drug duration of 10 to 15 months (except for the study by Chamie et al,^34^ which reported 5.1 months). Overall mean study drug treatment discontinuation due to placebo AEs was 3.9% (95% CI, 2.7%-5.2%) and due to disease recurrence was 27.9% (95% CI, 17.7%-36.3%). Furthermore, study drug dose reduction and interruption because of placebo AEs were only informed in 4 trials, with a mean of 7% (95% CI, 0.8%-14.8%) for study drug dose reduction and 11% (95% CI, 3.2%-18.8%) for study drug interruption (eTable 2 in the Supplement).

### Risk of Bias

Considerations of the risk of bias estimated for each trial are detailed in eFigure 4 in the Supplement. All trials had low risk of bias at randomization and masking, considering that both were inclusion criteria. A total of 60% of the studies had unclear risk of bias associated with incomplete data mainly for dose reduction and interruption. The funnel plot for publication bias showed no asymmetry (eFigure 5 in the Supplement).

## Discussion

In RCTs, placebo groups are designed to create a masked context in which results appreciated in the control group are attributable to the investigational setting to identify the real effect of the treatment assigned in the active therapy group.^36^ The need for masking in RCTs of both investigators and patients exposes the significance of placebo effect and placebo AEs in clinical practice. Therefore, RCTs, ideally double-blinded with a placebo group (control), have become a standard for clinical research.^37^

Addressing safety is a requirement for RCTs. Adverse events reported in both active therapy and placebo groups are a matter of interest. However, only a few studies have addressed this issue in patients with cancer. In a review of 37 RCTs of patients with different cancer types, Chvetzoff and Tannock^18^ reported AEs in 10% to 60% of the patients in the placebo group. Adjuvant clinical trials were excluded from that review. Common AEs were similar among the trials analyzed, and the authors described that there was an association in the type and rate of AEs between the treatment and placebo groups, just as was found in our study. In one study, Foster et al^12^ analyzed 2 RCTs among patients with advanced cancer in the adjuvant setting. The authors described that among the 446 patients included who received only placebo, 155 of 5234 placebo AEs reported were grade 3 to 5 according to the CTCAE. To our knowledge, there are no previous studies that analyzed the incidence of higher-grade placebo AEs in the adjuvant setting.

In the present study, rates of discontinuation due to placebo AEs were equal or greater than 5% in 4 trials. In addition, in different disease models, considerable rates of higher-grade placebo AEs were described, and discontinuations rates were reported in patients with fibromyalgia (9.5%), migraine (4.8%), and multiple sclerosis (2.2%).^38,39,40^ It needs to be considered that patients who were included in the discontinuation analysis because of disease recurrence rate were consequently excluded from the rate of discontinuation due to AEs. In some trials,^26,29^ high recurrence rates coincided with a high incidence of grade 3 to 4 placebo AEs. Patients experiencing disease recurrence might also report effects that could be considered as high-grade AEs in the context of a trial.

Median duration of placebo administration is a factor that should be taken into account when placebo AE reports are considered. In the studies analyzed, a trial^34^ with low median placebo exposure reported a low proportion of grade 3 to 4 placebo AEs, whereas studies^26,32,35^ with the highest median placebo exposure reported a high proportion of grade 3 to 4 placebo AEs.

Contextual factors associated with clinical trial participation may contribute to the heterogeneous frequency of severe placebo AEs, and different explanations should be considered. First, negative suggestions and expectations could be raised by the data provided during the informed consent process. To illustrate this point, in an RCT of aspirin as a treatment for unstable angina, a higher incidence of gastrointestinal irritation was reported in centers that specified its potential occurrence in the informed consent compared with research units that did not include that risk.^41^ The uncertainty of receiving an active treatment or placebo may also play a role in patients’ distress. Furthermore, entering into an RCT is often associated with greater exposure to a stressful environment (eg, interaction with symptomatic patients and cancer-information seeking), which can also be associated with negative suggestions among susceptible patients. We considered chemotherapy and hormonal therapy to be associated with different AE profiles compared with the type of drugs selected for our analysis. As a consequence, patients randomized to the placebo group in chemotherapy or hormonal therapy trials might be exposed to substantially different information in their consents, which would lead to a distinct profile of suggestions and expectations.

Second, a higher frequency of physician visits and medical examinations may be associated with an increased risk of overdiagnosis of AEs. Receipt of an unbeneficial diagnosis potentially leads to unnecessary additional examinations and may enhance iatrogenic damage.^42^ This last point should be carefully analyzed in the context of a patient who could experience a severe AE in which the double-blinding does not permit the physician to know if the patient is receiving the active drug or placebo.

Third, the rate of higher-grade AEs could be underestimated owing to underreporting. In a review^43^ of 3 RCTs that included patients with advanced cancer or were in the adjuvant setting, there was discordance in the appreciation of toxic effects, including cases in which patients described that they had “very much” toxicity. Some situations during AE reporting, such as the absence of predefined tools to standardize decisions regarding attribution and the existence of symptom-based categories defined by CTCAE criteria, could explain AE underestimation.^44^

Finally, the substantial differences between patients who underwent a local cancer treatment and healthy individuals might be considered as other factors that could explain the occurrence of AEs. For example, moderate to severe fatigue was reported in 17% to 23% of patients with early-stage non–small cell lung cancer who underwent local treatment.^20,45^ In all the included trials, the time from surgical resection to randomization was from 12 to 13 weeks. Therefore, the possible AEs overlapped by surgical procedures were the same for the entire population, and this bias could be mitigated.

According to the findings of this meta-analysis, placebo administration may be associated with severe AEs. This finding, frequently not included in the informed consents, should be known before making an autonomous decision of participating in an RCT. Although many patients may experience high-grade AEs after a local cancer treatment, the high rate of severe placebo AEs in RCTs suggests that the use of placebo in any situation in the adjuvant setting should be carefully considered.

Use of placebo may be proposed in certain circumstances, for instance, when a high incidence of subjective AEs is expected or if nonobjective end points are planned to determine factors associated with the active therapy group. New approaches should be considered by investigators, sponsors, regulatory authorities, and patient support groups. Concomitantly, patients should be informed about potential risks regarding randomization to the experimental drug or placebo, and regulatory authorities should not disregard the bioethical implications of RCTs.

### Limitations

Implications of these results may be limited to the heterogeneous population included in the analysis. There were unavailable data in the full text and supplemental appendixes of some trials, which could hamper a complete understanding of placebo AEs. For that reason, no association between sex, ethnic/racial characteristics, age, cancer stage, comorbidities, or placebo composition and the occurrence of grade 3 to 4 AEs could be made. Other potential contributing factors, such as the different local cancer treatment types that patients had undergone before entering the trial, could add heterogeneity to the population analyzed.

As a consequence of the limited number of studies analyzed and the high level of heterogeneity obtained, the incidence of high-grade placebo AEs should be considered with caution. Further investigation in this area with a large number of RCTs could help to obtain a better understanding of the potential factors that contribute to the placebo AEs.

## Conclusions

This systematic review and meta-analysis found that placebo administration was associated with a substantial proportion of grade 3 to 4 placebo AEs in double-blind RCTs in the adjuvant setting. Heterogeneity within included populations was observed.

## Notes

Supplement. **eMethods.** Search Strategy **eTable 1.** Descriptive Summary of Studies Included in the Analysis **eTable 2.** Extra Data From Selected Studies **eFigure 1.** Frequency of Grade 3-4 Adverse Events in the Treatment Groups vs Placebo Groups **eFigure 2.** Forest Plots of the Proportion of Grade 3-4 Adverse Events in the Placebo Groups Subdivided by Different Tumor Types **eFigure 3.** Correlation of Grade 3-4 Adverse Events Frequency Between the Treatment and Placebo Groups **eFigure 4.** Bias Across All Included Studies **eFigure 5.** Publication Bias
