# Supplementary material for: Incidence of Placebo Adverse Events in Randomized Clinical Trials of Targeted and Immunotherapy Cancer Drugs in the Adjuvant Setting: A Systematic Review and Meta-analysis
Source: JAMA Netw Open. 2018 Dec 7;1(8):e185617. doi: 10.1001/jamanetworkopen.2018.5617 (PMC6324542; doi:10.1001/jamanetworkopen.2018.5617)
Supplement: Supplement. — eMethods. Search Strategy eTable 1. Descriptive Summary of Studies Included in the Analysis eTable 2. Extra Data From Selected Studies eFigure 1. Frequency of Grade 3-4 Adverse Events in the Treatment Groups vs Placebo Groups eFigure 2. Forest Plots of the Proportion of Grade 3-4 Adverse Events in the Placebo Groups Subdivided by Different Tumor Types eFigure 3. Correlation of Grade 3-4 Adverse Events Frequency Between the Treatment and Placebo Groups eFigure 4. Bias Across All Included Studies eFigure 5. Publication Bias [file jamanetwopen-1-e185617-s001.pdf]

Supplementary Online Content

Chacón MR, Enrico DH, Burton J, Waisberg FD, Videla VM. Incidence of placebo adverse events in randomized clinical trials of targeted and immunotherapy cancer drugs in the adjuvant setting: a systematic review and meta-analysis. *JAMA Netw Open*. 2018;1(8):e185617. doi:10.1001/jamanetworkopen.2018.5617.

eMethods. Search Strategy

eTable 1. Descriptive Summary of Studies Included in the Analysis

eTable 2. Extra Data From Selected Studies

eFigure 1. Frequency of Grade 3-4 Adverse Events in the Treatment Groups vs Placebo Groups

eFigure 2. Forest Plots of the Proportion of Grade 3-4 Adverse Events in the Placebo Groups Subdivided by Different Tumor Types

eFigure 3. Correlation of Grade 3-4 Adverse Events Frequency Between the Treatment and Placebo Groups

eFigure 4. Bias Across All Included Studies

eFigure 5. Publication Bias

This supplementary material has been provided by the authors to give readers additional information about their work.

© 2018 Chacón MR et al. *JAMA Network Open*.

eMethods. Search Strategy

Medline (PubMed)

Separated searches were performed for each listed model. Limited to English, Clinical Trials and from 01/01/2000.

1. Breast: (adjuvant or consolidation or maintenance) AND (Breast neoplasm MeSH OR ((breast OR mammary) AND (cancer OR carcinoma OR malignan* OR neoplasm OR tumor))) AND ((placebo) OR (placebo*)) NOT animals

2. Lung: (adjuvant or consolidation or maintenance) AND (Lung neoplasm MeSH OR ((lung OR pulmonary) AND (cancer OR carcinoma OR malignan* OR neoplasm OR tumor))) AND ((placebo) OR (placebo*)) NOT animals

3. Melanoma: (adjuvant or consolidation or maintenance) AND (melanoma OR ((melanotic OR *melanotic) AND (cancer OR malignan* OR neoplasm OR tumor))) AND ((placebo) OR (placebo*)) NOT animals

4. Kidney: (adjuvant or consolidation or maintenance) AND (Kidney neoplasm MeSH OR ((kidney OR (clear AND cell) or renal) AND (cancer OR carcinoma OR malignan* OR neoplasm OR tumor))) AND ((placebo) OR (placebo*)) NOT animals

5. GIST: (adjuvant or consolidation or maintenance) AND (Gastrointestinal Stromal Neoplasm MeSH OR GIST OR ((gastric or gastrointestinal or intestinal) AND stromal) AND (cancer OR carcinoma OR malignan* OR neoplasm OR tumor)) AND ((placebo) OR (placebo*)) NOT animals

6. Liver (hepatocellular): (adjuvant or consolidation or maintenance) AND (Liver Neoplasm MeSH OR (hepatocellular or hepatic or liver) AND (cancer OR carcinoma OR malignan* OR neoplasm OR tumor)) AND ((placebo) OR (placebo*)) NOT animals

7. Uterine (uterine cervical and endometrial cancer): (adjuvant or consolidation or maintenance) AND (Endometrial Neoplasm MeSH OR Uterine Cervical Neoplasm MeSH OR (cervix or endometrial or endometrium or uterus or uterine) AND (cancer OR carcinoma OR malignan* OR neoplasm OR tumor)) AND ((placebo) OR (placebo*)) NOT animals

8. Ovarian: (adjuvant OR consolidation OR maintenance) AND (Ovarian Neoplasm MeSH OR (ovary OR gonad OR ovarian) AND (cancer OR carcinoma OR malignan* OR neoplasm OR tumor)) AND ((placebo) OR (placebo*)) NOT animals

9. Head and neck: (adjuvant OR consolidation OR maintenance) AND (Head Neck Neoplasms MeSH OR (larynx OR oropharynx OR hypopharynx OR hypopharyngeal OR nasopharynx OR nasopharyngeal OR cavum OR (upper AND aerodigestive) OR UADT OR lip OR tongue OR palatal OR palate OR pharynx OR laryngeal OR oropharyngeal OR (oral and cavity)) AND (cancer OR carcinoma OR malignan* OR neoplasm OR tumor)) AND ((placebo) OR (placebo*)) NOT animals

10. Bladder: (adjuvant or consolidation or maintenance) AND (Urinary Bladder Neoplasm MeSH OR (vesical OR bladder or urothelial) AND (cancer OR carcinoma OR malignan* OR neoplasm OR tumor)) AND ((placebo) OR (placebo*)) NOT animals

11. Prostate: (adjuvant or consolidation or maintenance) AND (Prostate Neoplasm MeSH OR (prostate OR prostatic) AND (cancer OR carcinoma OR adenocarcinoma OR malignan* OR neoplasm OR tumor)) AND ((placebo) OR (placebo*)) NOT animals

12. Testis: (adjuvant or consolidation or maintenance) AND (Germ Cell Neoplasm MeSH OR seminoma or non-seminoma OR (testicle OR testis or testicular or embryonal or embryonic or germ or germinal) AND (cancer OR carcinoma OR malignan* OR neoplasm OR tumor)) AND ((placebo) OR (placebo*)) NOT animals

13. Other sarcomas (GIST excluded): (adjuvant or consolidation or maintenance) AND (Sarcoma MeSH OR (mesoderm or mesenchymal or stromal) AND (malignan* OR neoplasm OR tumor)) AND ((placebo) OR (placebo*)) NOT animals

14. Esophageal and Gastric: (adjuvant or consolidation or maintenance) AND (Esophageal Neoplasm MeSH or Gastric Neoplasm MeSH OR (esophageal or esophagus or gastric or stomach or gastroesophageal or junction) AND (malignan* OR neoplasm OR tumor or carcinoma or cancer or adenocarcinoma)) AND ((placebo) OR (placebo*)) NOT animals

15. Pancreas: (adjuvant or consolidation or maintenance) AND (Pancreatic Neoplasm MeSH OR (pancreatic or pancreas) AND (malignan* OR neoplasm OR tumor or carcinoma or cancer or adenocarcinoma)) AND ((placebo) OR (placebo*)) NOT animals

© 2018 Chacón MR et al. *JAMA Network Open*.

16. Colorectal: (adjuvant or consolidation or maintenance) AND (Colorectal Neoplasm MeSH OR (colonic or colon or rectum or rectal) AND (malignan* OR neoplasm OR tumor or carcinoma or cancer or adenocarcinoma or polyp*)) AND ((placebo) OR (placebo*)) NOT animals

17. Biliary Tract, gallbladder and cholangiocarcinoma: (adjuvant or consolidation or maintenance) and (Cholangiocarcinomas MeSH OR Biliary Tract Neoplasms MeSH or (gallbladder or bile or duct or choledochus or Klatskin) And (malignan* OR neoplasm OR tumor or carcinoma or cancer or adenocarcinoma)) AND ((placebo) OR (placebo*)) NOT animals

18. GI Neuroendocrine tumors: (adjuvant or consolidation or maintenance) and (Neuroendocrine Tumors MeSH or NET or NEN or NEC or (neuroendocrine or (small and cell)) And (malignan* OR neoplasm OR tumor or carcinoma or cancer)) AND ((placebo) OR (placebo*)) NOT animals NOT lung

19. Brain: (adjuvant or consolidation or maintenance) and (Brain Neoplasm MeSH or (glioma) or (glioblastoma) or (astrocytoma) or ((brain or cerebellum or (spinal and cord)) And (malignan* OR neoplasm OR tumor or cancer))) AND ((placebo) OR (placebo*)) NOT animals

20. Anus: (adjuvant or consolidation or maintenance) and (Anus Neoplasms MeSH or ((anal or anus) And (malignan* OR neoplasm OR tumor or cancer))) AND ((placebo) OR (placebo*)) NOT animals

21. Vulva and vagina: (adjuvant or consolidation or maintenance) and (Vulvar Neoplasms MeSH or Vaginal Neoplasms MeSH or ((vulva or vulvar or vagina or vaginal) And (malignan* OR neoplasm OR tumor or cancer))) AND ((placebo) OR (placebo*)) NOT animals

22. Penis: (adjuvant or consolidation or maintenance) and (Penis Neoplasms MeSH or ((penis or penile) And (malignan* OR neoplasm OR tumor or cancer))) AND ((placebo) OR (placebo*)) NOT animals

eTable 1. Descriptive summary of studies included in the analysis

Parameter	Value
Number of studies	10
Mean number of patients per study (SD)	1,114.3 (586)
Total number of patients[a]	11,143
Total number of placebo-treated patients[a]	4,873 (43.7%)
Mean age (y) of placebo-treated patients[b] (SD)	55.9 (4.3)
Total number of active drug-treated patients[a]	6,270 (56.3%)
Mean age (y) of active drug-treated patients[b] (SD)	55.6 (4.2)
Placebo-treated men[b]	64%
Placebo-treated women[b]	36%
Route of administration	
Oral	6
Intravenous	3
Intramuscular	1
Mean of any grade adverse events in placebo-treated patients[a] (SD)	85.1% (9.1)
Mean discontinuation because of adverse events in placebo-treated patients[a] (SD)	3.9% (2)
Mean discontinuation because of disease recurrence in placebo-treated patients[b] (SD)	27.9% (15.8)
Most frequent grade 3-4 adverse events in placebo-treated patients[a] (Mean, SD)	
Hypertension	2.8% (2.2)
Fatigue	1% (0.9)
Diarrhea	0.8% (0.6)

Abbreviations: SD, standard deviation
[a] Patients included in safety analysis
[b] Total treated cohort

© 2018 Chacón MR et al. *JAMA Network Open*.

eTable 2. Extra data from selected studies

				Treatment Group versus Placebo Group, No. (%) of Patients		
Trials	Design	n[a] TG/PG	Time from Surgical Resection to Randomization	Dose Reduction because of AEs	Interruption because of AEs	Primary End Point
Eggermont et al,[26] 2015	Ipilimumab vs Placebo	471/474	12 weeks	NA	NA	RFS
Long et al,[27] 2017	Dabrafenib + Trametinib vs Placebo	435/432	12 weeks	167 (38) vs 11 (3)	289 (66) vs 65 (15)	RFS
Maio et al,[28] 2018	Vemurafenib vs Placebo	247/247	13 weeks	NS 155 (63) vs 38 (15)	NS 155 (63) vs 38 (15)	DFS
Eggermont et al,[29] 2018	Pembrolizumab vs Placebo	509/502	13 weeks	NR	NR	RFS
Vansteenkiste et al,[30] 2016	MAGE-A3 vs Placebo	1515/757	32 weeks[b]<br>12 weeks[c]	NR	NR	DFS
DeMatteo et al,[31] 2009	Imatinib vs Placebo	337/345	12 weeks	NS 52 (15) vs 10 (3)	NS 52 (15) vs 10 (3)	RFS
Haas et al,[32] 2016	Sunitinib/ Sorafenib vs Placebo	625/628/626	12 weeks	NR	NR	DFS
Motzer et al,[33] 2017	Pazopanib vs Placebo	766/762	12 weeks	408 (53) vs NR	NR	DFS
Chamie et al,[34] 2017	Girentuximab vs Placebo	431/424	12 weeks	NR	NR	DFS and OS
Ravaud et al,[35] 2016	Sunitinib vs Placebo	306/304	12 weeks	105 (34) vs 6 (2)	142 (46) vs 40 (13)	DFS

Abbreviations: TG, treatment group; PG, placebo group; NR, not reported; NA, not available; AEs, adverse events; DFS, disease-free survival; OS, overall survival; RFS, relapse-free survival; MAGE-A3, Melanoma-associated antigen 3.

[a]Patients included in the safety analysis

[b] Patients who received adjuvant chemotherapy

[c] Patients who did not receive adjuvant chemotherapy

© 2018 Chacón MR et al. *JAMA Network Open*.

eFigure 1. Frequency of Grade 3-4 Adverse Events in the Treatment Groups vs Placebo Groups

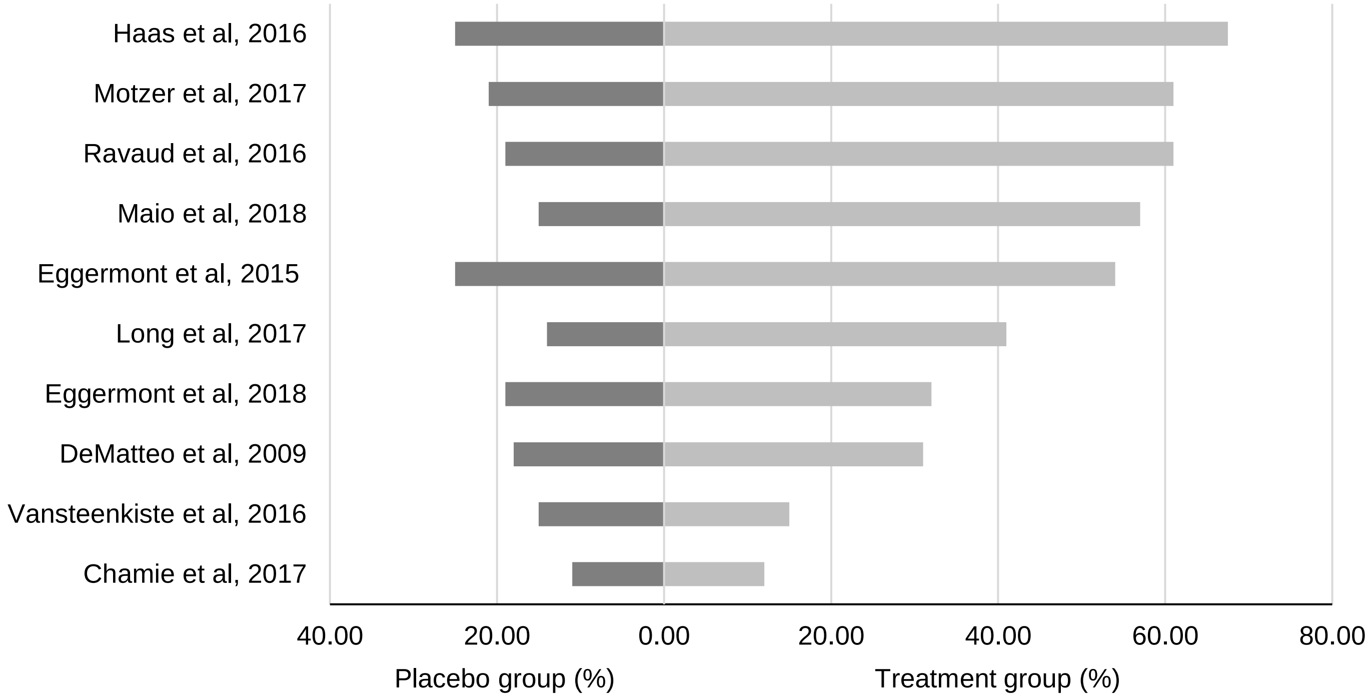

[a]Treatment group was calculated as the mean incidence of grade 3-4 adverse events in sunitinib and sorafenib patients.

© 2018 Chacón MR et al. *JAMA Network Open*.

eFigure 2. Forest Plots of the Proportion of Grade 3-4 Adverse Events in the Placebo Groups Subdivided by Different Tumor-Types

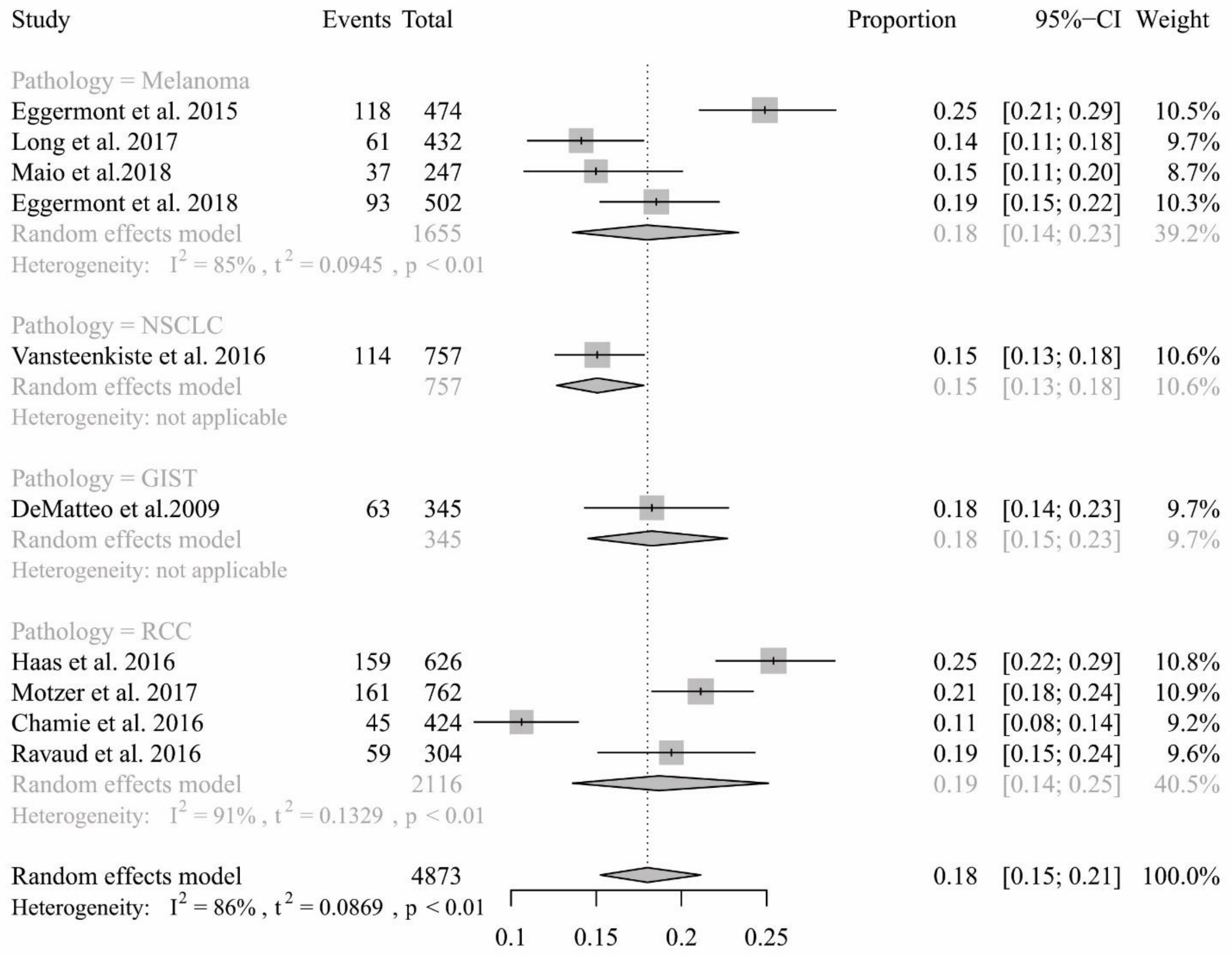

Abbreviations: AEs, adverse events; NSCLC, non–small cell lung cancer; GIST, gastrointestinal stromal tumor; RCC, renal cell carcinoma

© 2018 Chacón MR et al. *JAMA Network Open*.

eFigure 3. **Correlation of Grade 3-4 Adverse Events Frequency Between the Treatment and Placebo Groups**

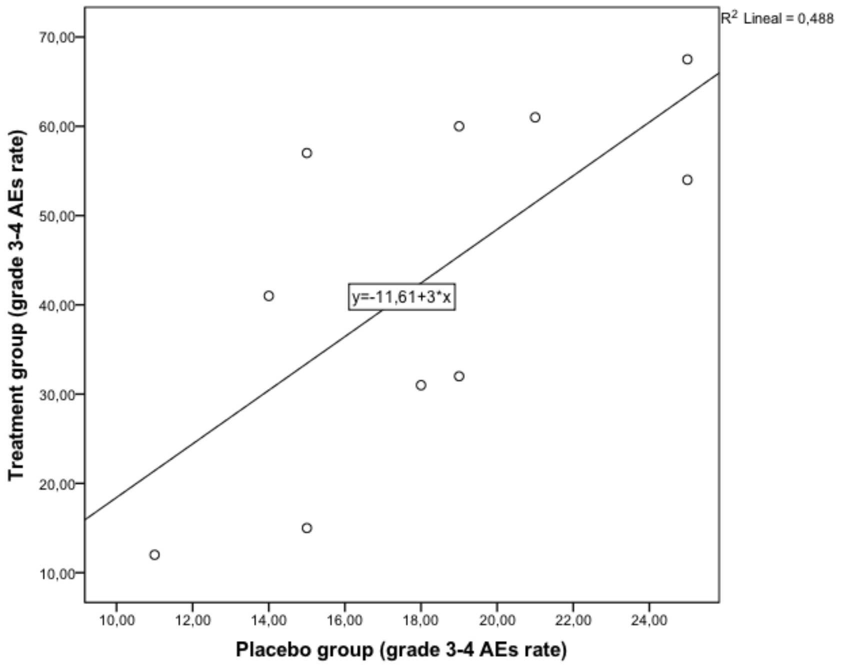

Abbreviations: AEs, adverse events
Each circle represents one study. Positive Spearman's correlation (ρ = 0.7; P = .03).

© 2018 Chacón MR et al. *JAMA Network Open*.

eFigure 4. Bias across all included studies

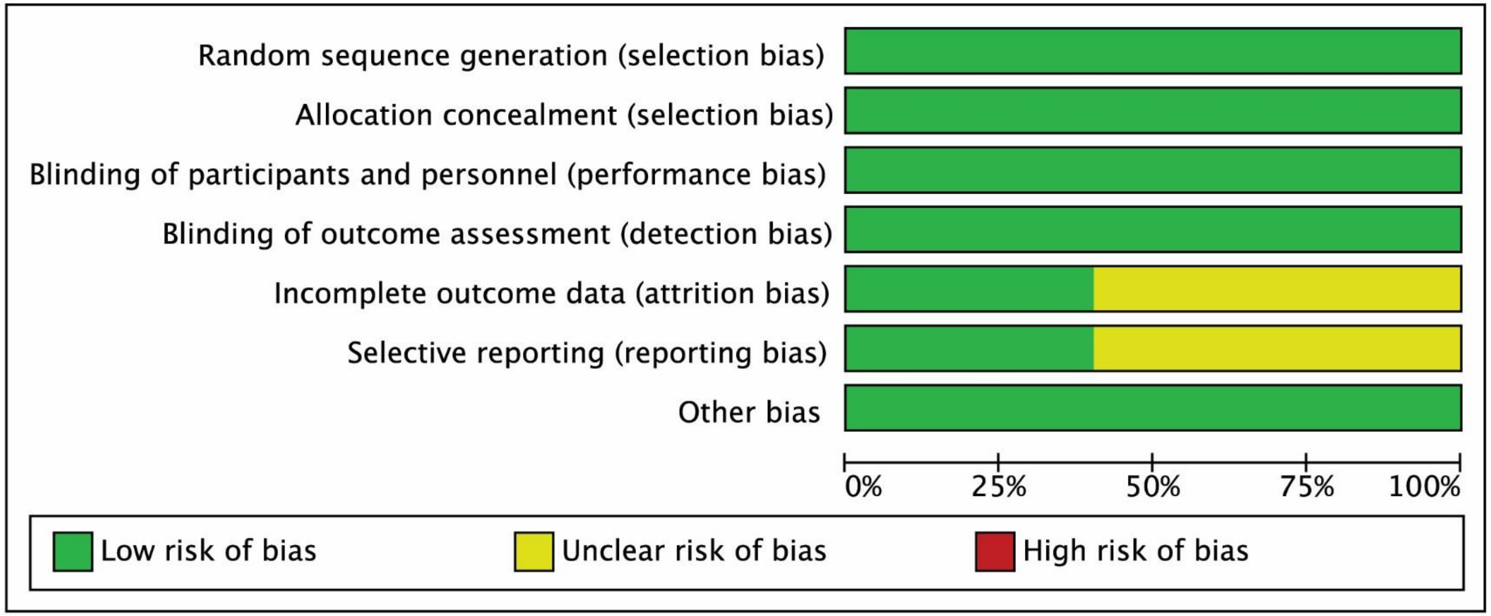

© 2018 Chacón MR et al. *JAMA Network Open*.

eFigure 5. Publication bias

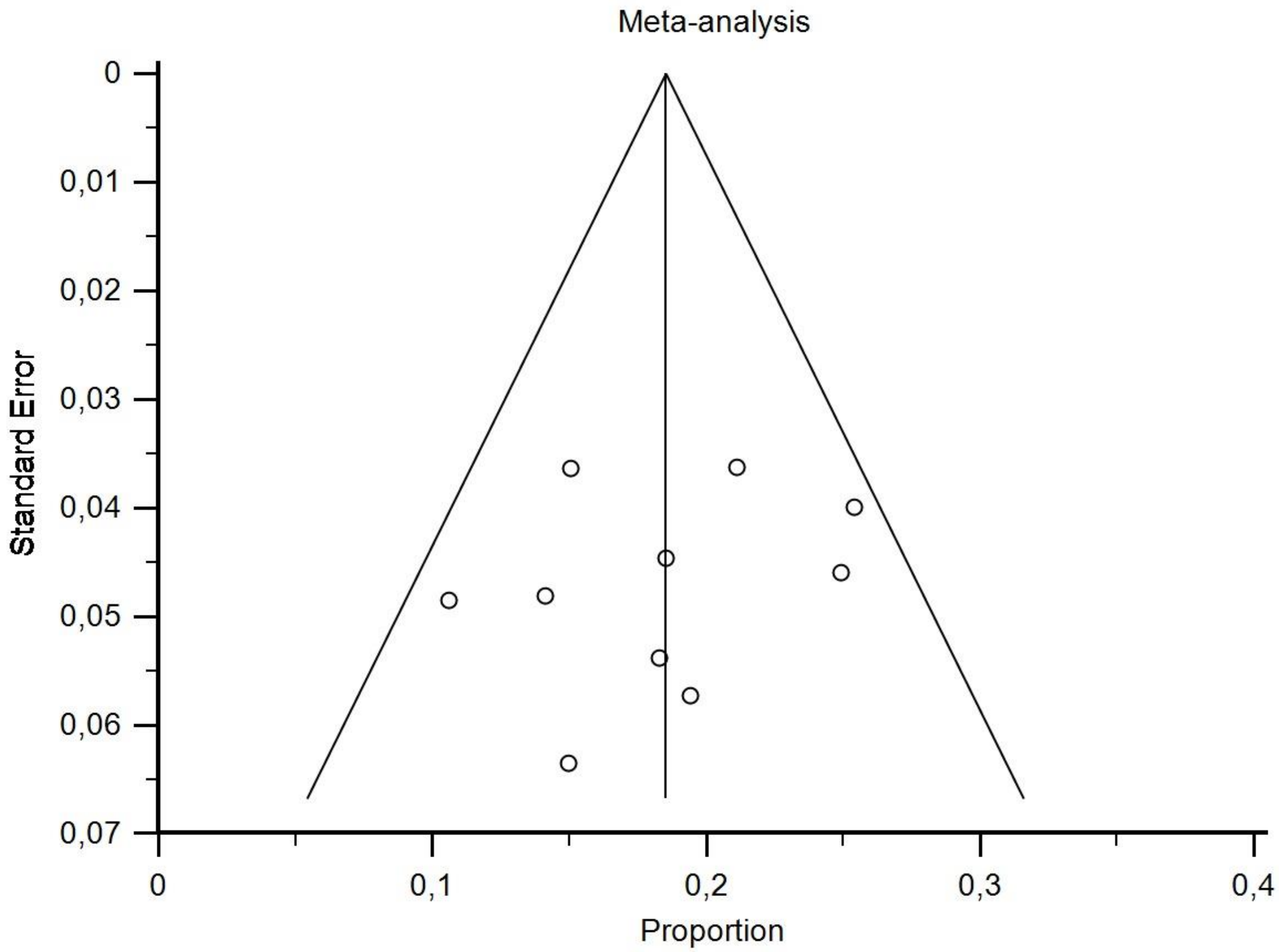

Publication bias funnel plot for grade 3-4 adverse event proportion in the placebo groups. Each dot represents a study. The solid vertical line represents the summary estimate proportion of grade 3-4 adverse events in the placebo groups using random-effect meta-analysis. The diagonal lines represent the 95% confidence limits around the summary for each standard error on the vertical axis.

© 2018 Chacón MR et al. *JAMA Network Open*.
